# Local changes in microtubule network mobility instruct neuronal polarization and axon specification

**DOI:** 10.1126/sciadv.abo2343

**Published:** 2022-11-04

**Authors:** Mithila Burute, Klara I. Jansen, Marko Mihajlovic, Tina Vermonden, Lukas C. Kapitein

**Affiliations:** ^1^ Cell Biology, Neurobiology and Biophysics, Department of Biology, Faculty of Science, Utrecht University, Padualaan 8, 3584 CH Utrecht, Netherlands.; ^2^ Department of Pharmaceutical Sciences, Utrecht Institute for Pharmaceutical Sciences (UIPS), Science for Life, Utrecht University, Universiteitsweg 99, 3508 TB Utrecht, Netherlands.

## Abstract

The polarization of neurons into axons and dendrites depends on extracellular cues, intracellular signaling, cytoskeletal rearrangements, and polarized transport, but the interplay between these processes during polarization remains unresolved. Here, we show that axon specification is determined by differences in microtubule network mobility between neurites, regulated by Rho guanosine triphosphatases (GTPases) and extracellular cues. In developing neurons, retrograde microtubule flow prevents the entry of the axon-selective motor protein Kinesin-1 into most neurites. Using inducible assays to control microtubule network flow, we demonstrate that local inhibition of microtubule mobility is sufficient to guide Kinesin-1 into a specific neurite, whereas long-term global inhibition induces the formation of multiple axons. We furthermore show that extracellular mechanical cues and intracellular Rho GTPase signaling control the local differences in microtubule network flow. These results reveal a novel cytoskeletal mechanism for neuronal polarization.

## Teaser

Polarized transport and axon specification are controlled by changes in microtubule network mobility between neurites.

## INTRODUCTION

**Fig. 1.**
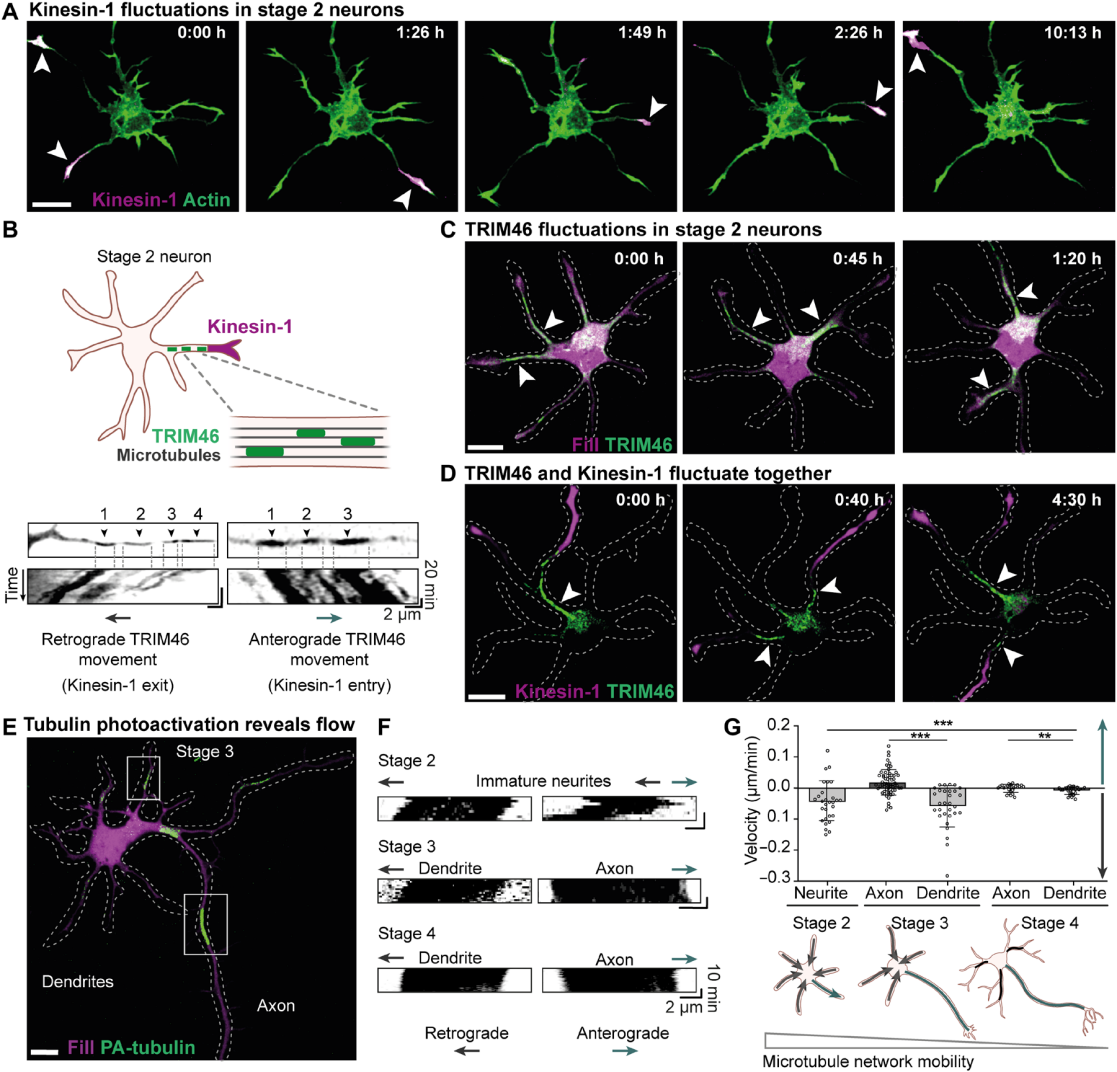
Neuronal polarization and axon specification correlate with reduced retrograde microtubule network flow. (**A**) Constitutively active Kinesin-1 [Kif5b(1–807)-GFP] (magenta) fluctuations between neurites of a stage 2 rat embryonic hippocampal neuron. White arrowheads indicate neurites with Kinesin-1 accumulation. (**B**) Directional movement of TRIM46 patches toward (retrograde) and away (anterograde) from the cell body. Top images show the start position, and kymographs show the movement of TRIM46 patches. (**C**) TRIM46 enrichment (green) fluctuates between different neurites of stage 2 neurons. White arrowheads indicate neurites with TRIM46 accumulation. (**D**) Appearance of TRIM46 (green) in a neurite (white arrowhead) is associated with Kinesin-1 (magenta) accumulation. (**E**) Stage 3 neuron expressing mRFP-fill (magenta) and photoactivatable tubulin (PA-tubulin). Boxes indicate PA-tubulin regions (green) in axon and dendrite used for kymographs in (F). (**F**) Kymographs representing movement of PA-tubulin regions at stage 2 to 4 neurons. (**G**) Top: Velocities of anterograde and retrograde microtubule flow (PA-tubulin movement) in stage 2 neurites (*n* = 29 regions), stage 3 dendrites (*n* = 30 regions) and axons (*n* = 62 regions), and stage 4 axons (*n* = 25 regions) and dendrites (*n* = 28 regions). ***P ≤ 0.001 and **P ≤ 0.01 (Mann-Whitney test for comparison between two groups). Analysis of variance (ANOVA) for comparison between five groups, *F*(4,145). Scale bars, 10 μm.

The proper functioning of neurons depends on their polarized organization into axons and dendrites. Neuronal polarization and axon specification require a delicate coordination between extracellular cues, intracellular signaling, cytoskeletal rearrangements, and selective transport into specific neurites, but the precise interplay between these processes is not known (*1*–*3*). Dissociated neurons that lack spatially defined extracellular cues also properly polarize (*4*). Several hours after plating, these neurons form multiple neurites (stage 2), of which one becomes the axon (stage 3), whereas the others later develop into dendrites (stage 4). In developing neurons, the selective accumulation of the microtubule-based motor protein Kinesin-1 is an early marker for the future axon (*5*–*7*), and several polarity factors depend on Kinesin-1 for transport into the axon (*8*–*11*). However, before accumulating in the future axon, Kinesin-1 first transiently enriches in a small and alternating subset of neurites during stage 2, representing a form of transient polarization (Fig. 1A and movie S1) (*5*, *7*, *12*). Various models have been proposed for this polarized transport of Kinesin-1 (*3*, *6*, *13*–*16*), but none of these explain how Kinesin-1 cycles between neurites and how this cycling can be controlled to more precisely define the future axon.

## RESULTS AND DISCUSSION

### Neuronal polarization and axon specification correlate with reduced retrograde flow of the microtubule network

To understand the mechanisms that control the polarized transport of Kinesin-1, we searched for changes in the microtubule cytoskeleton that were associated with changes in the selective accumulation of Kinesin-1. Live-cell imaging of the microtubule-associated protein TRIM46, an early axon marker (*17*), revealed that it also cycled between neurites in stage 2 neurons and its selective localization at the base of specific neurites was strongly correlated with Kinesin-1 transport into that neurite (Fig. 1, B to D; fig. S1; and movie S2). In addition, faster imaging revealed that various microtubule-associated patches of TRIM46 underwent collective anterograde or retrograde motility at 0.04 to 0.3 μm/min (Fig. 1B and movie S3). Notably, anterograde or retrograde movement of TRIM46 corresponded with Kinesin-1 entry or exit from neurites, respectively (Fig. 1D; fig. S2, A to C; and movie S4).

The synchronized movement of TRIM46 within neurites suggests that the entire microtubule network could be moving collectively in the neurites of stage 2 neurons. We therefore monitored the microtubule network dynamics using local photoactivation of tubulin (*18*). This revealed that the microtubule network moved as a whole in neurites of stage 2 and 3 neurons, featuring both retrograde and anterograde motility (Fig. 1, E and F). During subsequent neuron maturation, the overall flow of the microtubule network and TRIM46 dampened (Fig. 1, E to G; fig. S1; and movies S5 and S6) (*19*). To understand whether the direction of microtubule network flow determined the polarized transport of Kinesin-1 and thereby instructed axon specification, we first compared the direction of network flow between different neurites. The microtubule network mostly moved retrogradely in stage 2 neurites, but with occasional reversals in a subset of neurites (Fig. 1F). In stage 3 neurons, microtubule flow in the axon strongly dampened, whereas retrograde flow was retained in the dendrites (Fig. 1G), suggesting that the absence of microtubule retrograde flow could be necessary for neuron polarization and axon specification. To directly test the correlation between microtubule network flow and Kinesin-1, we next combined local tubulin photoactivation with Kinesin-1 imaging. This revealed that exit of Kinesin-1 correlated with retrograde microtubule flow, whereas Kinesin-1 was retained in neurites without retrograde microtubule flow (fig. S2A and movie S7).

### Local inhibition of retrograde microtubule flow is sufficient to instruct neuronal polarization

**Fig. 2.**
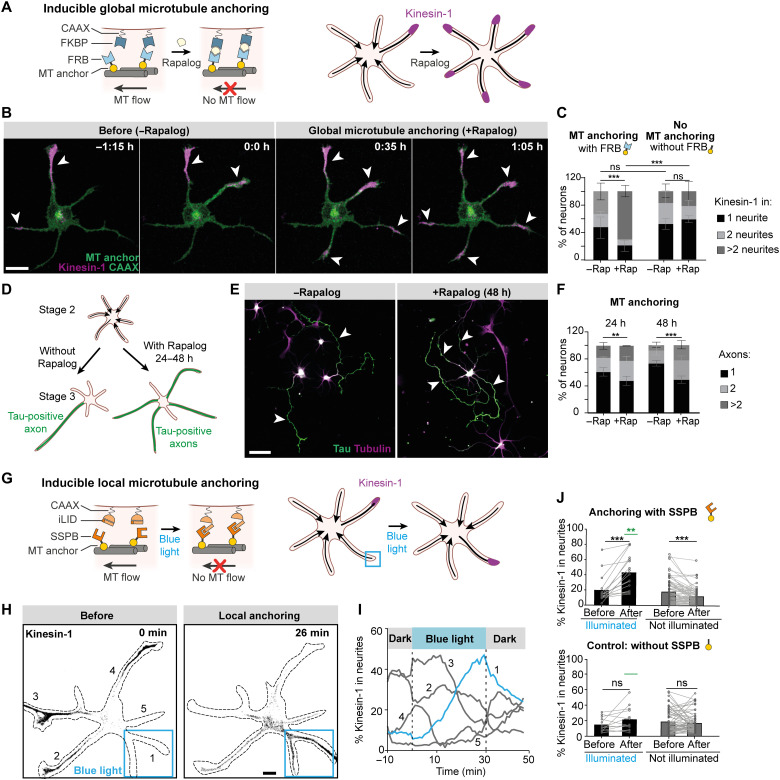
Local inhibition of retrograde microtubule flow is sufficient to instruct neuronal polarization. (**A**) Global microtubule (MT) anchoring assay. Rapalog induces FKBP-FRB dimerization and globally anchors microtubules to the cell membrane. (**B**) After global microtubule anchoring, Kinesin-1 [Kif5b(1–353)-GCN4-mCherry] (magenta) fluctuations between neurites stop and Kinesin-1 enters all neurites. Scale bar, 10 μm. (**C**) Percentage of stage 2 neurons with Kinesin-1 accumulation in 1, 2, or >2 neurites. With FRB module: *n* = 75 neurons without rapalog and *n* = 101 neurons with rapalog from three independent experiments. Without the FRB module: *n* = 79 neurons without rapalog and *n* = 79 neurons with rapalog from three independent experiments. Error bars represent SD. ***P ≤ 0.001 and ns (nonsignificant), *P* > 0.05 (chi-square test). (**D**) Long-term global microtubule anchoring assay. (**E**) Example of neurons forming single or multiple axons without or with microtubule anchoring, respectively. Scale bar, 50 μm. (**F**) Quantification of long-term anchoring experiments. Twenty-four-hour condition: Number of neurons without rapalog (*n* = 170) and with rapalog (*n* = 188) from three independent experiments. Forty-eight-hour condition: Number of neurons without rapalog (*n* = 208) and with rapalog (*n* = 225) from three independent experiments. Error bars represent SD. **P ≤ 0.01 and ***P ≤ 0.001 (chi-square test). (**G**) Light-induced local microtubule anchoring assay. (**H**) Kinesin-1 is relocated from neurites 2, 3, and 4 to neurite 1 upon local blue light illumination. Scale bar, 10 μm. (**I**) Time traces of Kinesin-1 accumulation in neurites of the neuron shown in (H). The blue trace represents the neurite illuminated by blue light. (**J**) Quantification of Kinesin-1 accumulation before and after blue light activation in neurites activated for 40 to 60 min and non-illuminated neurites in the same neurons. Top/bottom: Microtubule anchoring with/without SSPB module. Blue light activated pairs (*n* = 16/*n* = 13) and nonactivated pairs (*n* = 76/*n* = 58). Bars represent median values. Green lines represent comparison between illuminated neurites with and without SSPB module. ***P ≤ 0.001 (two-tailed Wilcoxon matched pair test) and **P ≤ 0.01 (Mann-Whitney test).

These results indicate that retrograde microtubule flow prevents entry of Kinesin-1 and suggest that Kinesin-1 will enter all neurites if the microtubule network flow is stopped throughout the neuron. To test this, we developed an assay to globally oppose the microtubule flow in stage 2 neurites by anchoring the microtubules to the plasma membrane (Fig. 2A). In this approach, an FKBP domain was targeted to the plasma membrane (FKBP-CAAX), and addition of the small-molecule rapalog would trigger interaction with its binding partner FRB fused to a microtubule-interacting protein known to tightly interact with microtubules (Kif5b-rigor) (*20*). We validated this approach in non-neuronal cells and found that microtubules robustly enriched near the plasma membrane within 40 min after rapalog addition and displayed reduced movements (fig. S3, A and B, and movies S8 and S9). In addition, we confirmed that rapalog addition to neurons expressing the two constructs to anchor microtubules resulted in arrested flow of TRIM46 in neurons (fig. S3, C and D). Notably, we found that within 1 hour after rapalog addition to globally oppose microtubule flow, Kinesin-1 had lost its polarized distribution and had instead accumulated in multiple neurites (Fig. 2, B and C, and movie S10). Similar results were obtained when we used another microtubule anchor (APC2-C; fig. S3, E to G). These results demonstrate that retrograde microtubule flow opposes Kinesin-1 entry and that differences in microtubule flow control the polarized distribution of Kinesin-1 in developing neurons.

To test whether long-term inhibition of microtubule retrograde flow in neurites triggers them to develop into axons, we globally anchored microtubules for extended periods of time (24 and 48 hours) and determined the number of axons per neuron. This revealed that, by opposing microtubule retrograde flow, the number of axons per neuron significantly increased (28% versus 52% neurons with >1 axon in control versus anchoring conditions) (Fig. 2, D to F). Thus, inhibition of microtubule retrograde flow is sufficient to trigger axon formation.

Our finding that global inhibition of microtubule network mobility triggers Kinesin-1 accumulation in all neurites suggests that local control over microtubule flow could control the polarized transport of Kinesin-1 in stage 2 neurons. To test whether local anchoring of microtubules is sufficient to guide the selective transport of Kinesin-1 into a specific neurite, we locally anchored the microtubule network to the plasma membrane using light-inducible dimerization. We exchanged the modules for chemically induced dimerization for modules that enable light-induced dimerization, i.e., iLID and SSPB (Fig. 2G) (*21*). Within 30 min after light-induced local anchoring of the microtubule network, Kinesin-1 transport was robustly guided into the selected stage 2 neurite, along with enrichment of an early axonal marker, TRIM46 (Fig. 2, H to J; fig. S4; and movie S11). In contrast, when the microtubule anchor was expressed without FRB or SSPB, addition of rapalog or blue light illumination did not alter the polarized transport of Kinesin-1 (Fig. 2, C and J, and fig. S5). These results directly demonstrate that local reduction of retrograde microtubule flow is sufficient to control Kinesin-1 polarized transport, which is necessary for axon specification.

### Rac1 and Cdc42 control microtubule network flow and neuronal polarization

**Fig. 3.**
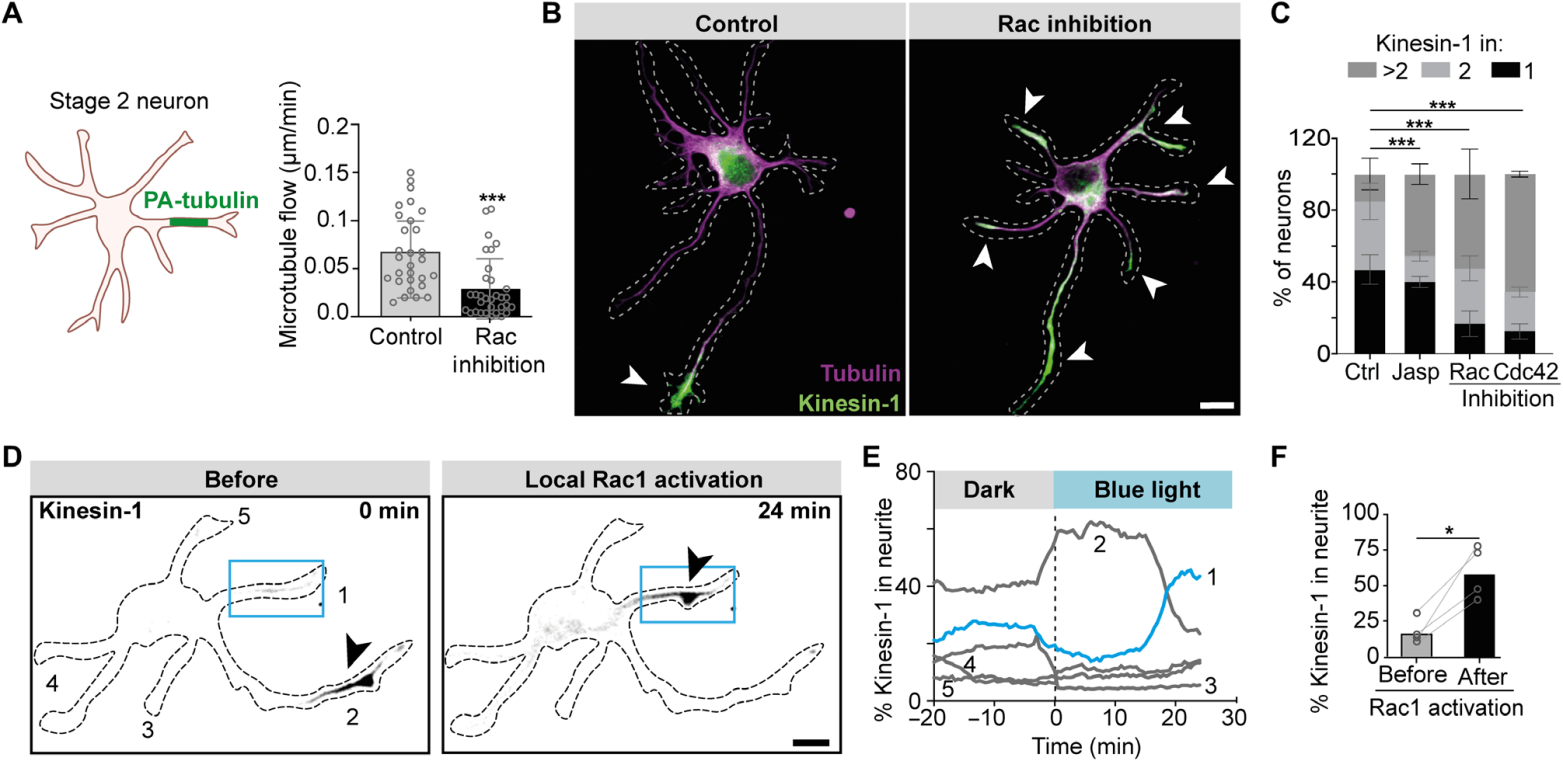
Rac1 and Cdc42 control microtubule network flow and neuronal polarization. (**A**) Quantification of speed of PA-tubulin regions in stage 2 neurons after Rac inhibition. Control (*n* = 29) and Rac inhibition (*n* = 32). (**B**) Images of stage 2 neurons expressing Kinesin-1. Kinesin-1 (green) accumulated in a single neurite in control condition but in multiple neurites after Rac inhibition. (**C**) Percentage of stage 2 neurons with Kinesin-1 accumulation in 1, 2, or >2 neurites. Control (*n* = 221), Rac inhibitor (*n* = 78), Cdc42 inhibitor (*n* = 77), and Jasplakinolide (*n* = 78) from three to six independent experiments. Error bars represent SD. ***P ≤ 0.001 (chi-square test). (**D**) Local photoactivation of Rac1 (blue box) changes selective accumulation of Kinesin-1 (gray) from neurite 2 to neurite 1 (black arrowheads). (**E**) Time trace of percentage of Kinesin-1 accumulation within neurites during Rac1 photoactivation. The blue trace indicates neurite 1 in which Rac1 was activated, and gray traces indicated nonactivated neurites as numbered in (D). (**F**) Percentage of Kinesin-1 accumulation in neurites before and after Rac1 photoactivation in stage 2 neurons. Bars indicate the median value. *P ≤ 0.05 (paired *t* test). (B and D) Scale bars, 10 μm.

We next searched for upstream regulators that could control the spatial differences in microtubule network flow. Various Rho family guanosine triphosphatases (GTPases), such as Rac1 and Cdc42, have been implicated in neuronal polarity and axon formation (*22*–*24*). These proteins are key regulators of the actin cytoskeleton, which is crucial during neuron development and axon specification, and could underlie the microtubule network flow that we observed (*6*, *25*). We therefore examined whether inhibition of the axon-specifying GTPase Rac1 affects microtubule network flow. Rac inhibition caused a threefold reduction in the speed of microtubule flow in stage 2 neurons (Fig. 3A). Consistently, inhibition of Rac1 or Cdc42 triggered the entry of Kinesin-1 into multiple neurites (Fig. 3, B and C, and fig. S6). Furthermore, stabilization of the actin network by jasplakinolide mimicked the effect of Rho GTPase inhibitors and also resulted in Kinesin-1 entry into multiple neurites (Fig. 3C), suggesting that microtubule network mobility depends on a dynamic actin cytoskeleton.

Next, we tested whether local activation of Rac1 was sufficient to alter microtubule flow and drive Kinesin-1 into a specific neurite. Local activation of Rac1 using photoactivatable Rac1 (PA-Rac1) was sufficient to guide Kinesin-1 into a single neurite (Fig. 3, D to F, and movie S12). We furthermore observed that local activation of Rac1 induced anterograde microtubule flow in the selected neurite and simultaneously triggered retrograde flow in all other neurites (fig. S7). This could explain why retrograde flow is reduced in all neurites in neurons without active Rac1 (Fig. 3A). Together, these results demonstrate that the Rho GTPases Rac1 and Cdc42 regulate microtubule network flow and thereby control the polarized accumulation of Kinesin-1 and subsequent axon specification.

### Microtubule network mobility is necessary for neuronal polarization driven by extracellular cues

**Fig. 4.**
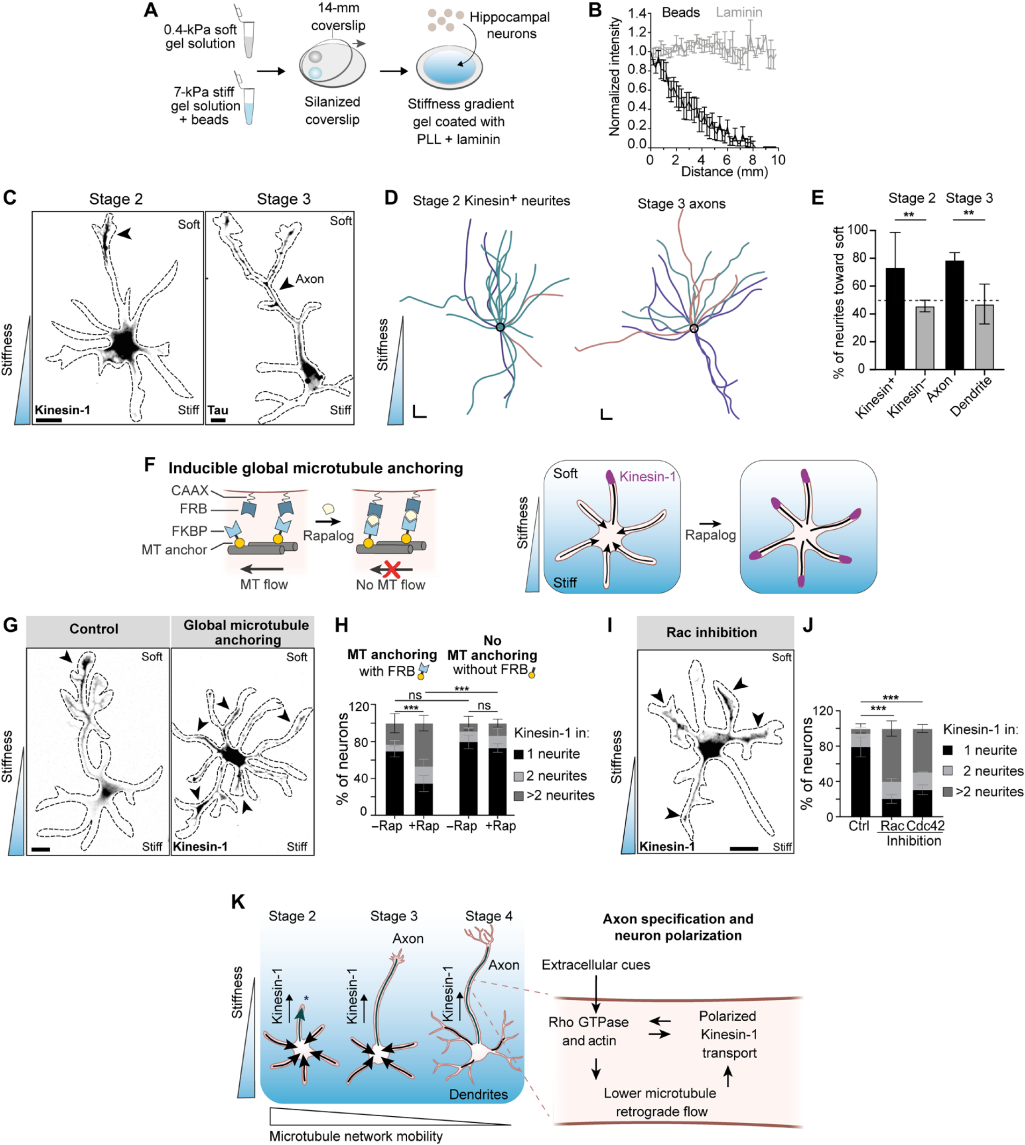
Microtubule network mobility is necessary for neuronal polarization driven by extracellular cues. (**A**) Preparation of stiffness gradient using premixes for stiff and soft gels. PLL, poly-l-lysine. (**B**) Bead density and laminin intensity on the stiffness gradient hydrogels (*n* = 11). Error bars: SEM. (**C**) Stage 2 and stage 3 neurons grown on stiffness gradients. Left: Stage 2 neuron expressing Kinesin-1. Right: Stage 3 neuron labeled for the axon marker Tau. Arrowheads mark neurite with Kinesin-1 accumulation or Tau-positive axon, both growing toward the softer matrix. Scale bars, 10 μm. (**D**) Orientation of traces of stage 2 neurites with Kinesin-1 and stage 3 axons on stiffness gradients. Black circles represent position of cell bodies. Colors represent traces from independent experiments. Scale bars, 20 μm (*x* and *y*). (**E**) Percentage of stage 2 neurites with and without Kinesin-1 accumulation (*n* = 22 and 62 neurites) and stage 3 axons and dendrites (*n* = 22 and 46) directed toward the soft matrix on stiffness gradients. **P ≤ 0.01 (chi-square test). (**F**) Rapalog-induced global microtubule anchoring assay on stiffness gradient. (**G**) Kinesin-1 accumulation with or without global microtubule anchoring. Scale bar, 10 μm. (**H**) Kinesin-1 accumulation after global microtubule anchoring. With FRB module: *n* = 80 neurons without rapalog and *n* = 104 neurons with rapalog from three independent experiments. Without the FRB module: *n* = 97 neurons without rapalog and *n* = 134 neurons with rapalog from four independent experiments. Error bars represent SD. ***P ≤ 0.001 and ns, *P* > 0.05 (chi-square test). (**I**) Stage 2 neuron expressing Kinesin-1 (gray), grown on a stiffness gradient matrix and treated with a Rac inhibitor. Scale bar, 20 μm. (**J**) Kinesin-1 accumulation in different conditions. Control (*n* = 112), Rac inhibitor (*n* = 128), and Cdc42 inhibitor (*n* = 47) from three independent experiments. Error bars: SD. ***P ≤ 0.001 (chi-square test). (**K**) Summary of the interplay between substrate elasticity, microtubule network mobility, and Kinesin-1 accumulation for axon specification and neuron polarization during development.

Within tissues, extracellular cues often guide the polarization of neurons along a predefined axis (*2*). To examine the importance of microtubule flow during such externally biased polarization, we set out to establish a robust model system for guided polarization by extracellular matrix cues. Recent work revealed that mechanical properties of brain tissue, e.g., stiffness gradients, control important processes of brain development, including neural crest migration and axon growth (*26*, *27*). Furthermore, mechanical properties of the extracellular matrix are known to control intracellular Rho GTPase signaling and dynamics of the actin cytoskeleton (*28*), which led us to hypothesize that microtubule network flow and subsequent axon specification could be controlled by mechanical properties of the extracellular matrix. We therefore monitored polarized transport of Kinesin-1 and axon specification in neurons grown on laminin-coated gels with a stiffness gradient varying from 0.4 to 7 kPa (Fig. 4, A and B). We used a recently introduced method to generate gradient gels in which the density of embedded fluorescent beads reports the local stiffness and can be used to map the stiffness landscape surrounding each neuron (fig. S8) (*29*). Dissociated rat hippocampal neurons were plated on these gels directly upon dissection. We found that Kinesin-1 was enriched in neurites growing toward the softer side of the matrix in ~73% of stage 2 neurons, whereas ~79% of axons of stage 3 neurons were directed toward the softer side of the matrix (Fig. 4, C to E, and figs. S9 and S10). These results demonstrate that polarized transport of Kinesin-1 and axon formation can be guided by stiffness gradients.

To test whether the polarized transport of Kinesin-1 during externally guided polarization is controlled by microtubule network flow, we used the global microtubule anchoring assay. Upon rapalog-induced microtubule anchoring, the selective accumulation of Kinesin-1 in neurites growing toward softer matrix was lost and Kinesin-1 was instead enriched in multiple neurites (Fig. 4, F to H). In control experiments that used a microtubule anchor lacking the heterodimerization domain, the distribution of Kinesin-1 remained polarized (Fig. 4H). Furthermore, global inhibition of Rac1 or Cdc42 activity also disturbed the selective entry of Kinesin-1 in neurons on stiffness gradients (Fig. 4, I and J). These results demonstrate that extracellular cues control neuronal polarization by controlling microtubule network flow to affect polarized transport.

In this work, we uncovered a novel cytoskeletal mechanism underlying neuronal polarization and axon specification (Fig. 4K). We found that, in developing neurons, most neurites have a microtubule network that undergoes retrograde flow, which prevents the entry of Kinesin-1 required for axon specification. Kinesin-1 only enters neurites without retrograde flow, and local anchoring of the microtubule network can be used to control polarization by guiding Kinesin-1 into specific neurites. When retrograde flow is opposed by global anchoring of the microtubule network, Kinesin-1 accumulates in all neurites and neurons do not polarize, not even in the presence of extracellular guidance cues. Thus, proper polarization depends on differences in microtubule network mobility between different neurites (*30*). The observed changes in microtubule network mobility (~0.1 μm/min) are too subtle to directly explain the complete loss of Kinesin-1 from a neurite, because Kinesin-1 (~1 μm/s) itself moves much faster and should, in principle, be able to outrun the retrograde microtubule movement. Hence, we speculate that the activity of Kinesin-1 itself could be regulated by the Rho GTPases that also control microtubule network mobility during neuron polarization (Fig. 4K). A recent study in the nematode *Caenorhabditis elegans* revealed the importance of an Ankyrin/UNC119/CRMP complex in controlling microtubule sliding during polarization (*31*), suggesting a dynamic interplay between reduced microtubule flow and formation of the axon initial segment.

Our results demonstrate that microtubule retrograde flow is controlled by Rho GTPases that control cytoskeletal dynamics, and that inhibition of the Rho GTPases Rac1 and Cdc42 or stabilization of actin leads to Kinesin-1 accumulation in all neurites. These results suggest that the actin cytoskeleton contributes to microtubule network mobility, for example, through coupling of microtubules to actin retrograde flow, most likely augmented by forces of (cortically anchored) microtubule-based motors and forces resulting from microtubule polymerization against neurite tips (*32*). Earlier work has shown that increased stiffness of the extracellular matrix stimulates the activity of myosin II, a driver of actin retrograde flow (*33*, *34*), which could provide a mechanistic explanation for our observation that neuronal polarity can be guided by stiffness gradients. Specifically, a stiffer matrix would increase actin retrograde flow and result in retrograde microtubule flow that prevents Kinesin-1 entry, thereby biasing Kinesin-1 entry toward the neurites growing on softer matrix.

In summary, our work reveals a cellular cascade by which extracellular (mechanical) cues can specify the polarity axis of neurons.

## MATERIALS AND METHODS

### DNA constructs

All constructs used in this study, as well as their source, are listed in Table 1. New constructs generated for this study were cloned into the mammalian expression vector pβactin-16-pl (chicken β-actin promoter) (*35*) by conventional molecular cloning and polymerase chain reaction (PCR)–based cloning. For all motor constructs, the human *Kif5b* sequence (P33176) was used. To avoid dimerization of the motile kinesin constructs with K560Rigor, we replaced the dimerization domain of Kinesin-1 with a GCN4 leucine zipper (LEDKIEELLSKIYHLENEIARLKKLIGEI) for the K353-GCN4-GFP and K353-GCN4-mCherry constructs (*35*). Synthetic, 29–amino acid GGGS linkers were added between domains as indicated by “L” in the details of the constructs in Table 1 (*36*). To anchor microtubules to the plasma membrane, we introduced the G234A mutation in hKif5b to create K560Rigor (*37*). The sequence for mNeongreen was provided by Allele Biotechnology (*38*), and the fluorophore was flanked by short linker sequences. mVenus and SspB(micro) domains were derived from pB80-KIF1A(1-365)-mVenus-SspB(micro) (*38*). The CAAX motif CVIM was derived from Venus-iLID-CAAX. The FKBP-FRB heterodimerization modules were described previously in (*20*).

**Table 1.** Table of DNA constructs. L, linker.

**Constructs**	**Reference**	**Details**
APC2-C-RFP-FRB	(*41*)	
CAAX-iLID	Addgene (#85680)	
FKBP-mCherry-CAAX	This paper	2xFKBP-L-mCherry- CAAX
K353-GCN4-GFP	This paper	hKif5b(1–353)-GCN4- L-GFP
K353-GCN4-mCherry	This paper	hKif5b(1–353)-GCN4- L-mCherry
K560-GFP	This paper	hKif5b(1–560)-L-GFP
K560Rigor-mNeon-FRB	This paper	hKif5b(1–560) Rigor- 2xmNeongreen-FRB
K560Rigor-HA	This paper	hKif5b(1–560) Rigor-L-2xHA
K560Rigor-HA-FRB	This paper	hKif5b(1–560) Rigor-L-2xHA-L-FRB
K560Rigor-mVenus	This paper	hKif5b(1–560) Rigor-L-mVenus
K560Rigor-mVenus- SspBmicro	This paper	hKif5b(1–560) Rigor-L-mVenus-L- SspB(micro)
K807-GFP-FRB	(*20*)	
K807-mCherry-FKBP	This paper	hKif5b(1–807)-L- mCherry-L-FKBP
LifeAct-mCherry	This paper	
mRFP-Fill	(*42*)	
PA-tubulin	(*42*)	PA-GFP tubulin
PA-Rac1	Addgene (#22024)	
TRIM46-GFP	(*17*)	
TRIM46-mCherry	(*17*)	

### Animals, neuron culture, and transfection

All animal experiments were approved by the Dutch Animal Experiments Committee (Dier Experimenten Commissie) (license number AVD1080020173404) and were in line with the institutional guidelines of Utrecht University. All animal experiments were performed in compliance with Dutch law (Wet op de Dierproeven, 1996) and European regulations (Directive 2010/63/EU). Pregnant Wister rats (Janvier), which were not involved in any previous experiments and were ≥10 weeks of age, were used in this study. For the primary neuron culture, cells were derived from hippocampi of embryonic day 18 (E18) pups (both genders). Hippocampi were dissociated using a method involving both enzymatic and mechanical dissociation (*39*). After dissociation, neurons were first electroporated (see below) or plated directly at a density of 50,000 cells per well in a 12-well plate on coverslips coated with poly-l-lysine (37.5 μg/ml; Sigma-Aldrich) and laminin (1.25 μg/ml; Roche). The primary cultures were maintained in Neurobasal medium (NB; Gibco) supplemented with 2% B27 (Gibco), 0.5 mM glutamine (Gibco), 15.6 μM glutamate (Sigma-Aldrich), and 1% penicillin/streptomycin (Gibco) (from now on referred to as full medium) at 37°C and 5% CO_2_ (Table 2).

**Table 2.** Table of chemicals. DMSO, dimethyl sulfoxide; PBS, phosphate-buffered saline; TEMED, *N*,*N*,*N*′,*N*′-tetramethylethylenediamine.

**Chemicals**	**Source**	**Reference**
16% Paraformaldehyde EM grade	Electron Microscopy Sciences	Catalog #15710
2% Bis-acrylamide solution	Fisher Scientific	Catalog #BP1404
3-(Trimethoxysilyl)propyl methacrylate	Sigma-Aldrich	Catalog #M6514
40% Acrylamide solution	Sigma-Aldrich	Catalog #A4058
8% Glutaraldehyde solution	Sigma-Aldrich	Catalog #G7526
Acetic acid (J. T. Baker)	Fisher Scientific	Catalog #15578364
Alexa Fluor Plus 405 phalloidin	Thermo Fisher Scientific	Catalog #A30104
Ammonium persulfate	Sigma-Aldrich	Catalog #215589
B27 supplement	Gibco	Catalog #17504001
Bovine serum albumin (BSA)	Carl Roth	Catalog #8076.3
DMSO, anhydrous	Thermo Fisher Scientific	Catalog #D12345
EHT1864 (Rac inhibitor)	Tocris	Catalog #872
Ethanol	Fisher Scientific	Catalog #15568474
FluoSpheres carboxylate- modified microspheres, 0.2 μm, dark red fluorescent (660/680), 2% solids	Thermo Fisher Scientific	Catalog #F8807
FuGENE 6	Promega	Catalog #E2692
l-Glutamic acid	Sigma-Aldrich	Catalog #G1251
l-Glutamine	Gibco	Catalog #25030-081
Hydrazine hydrate	Sigma-Aldrich/ Merck	Catalog #225819
Laminin	Roche	Catalog #1124321700
Jasplakinolide	Tocris/ Bio-Techne	Catalog #2792
Leibovitz’s L-15 medium	Thermo Fisher Scientific	Catalog #11415064
Lipofectamine 2000	Thermo Fisher Scientific	Catalog #11668019
ML141 (Cdc42 inhibitor)	Tocris	Catalog #4266
Neurobasal medium	Gibco	Catalog #21103049
PBS	Lonza	Catalog #BE17-517Q
Penicillin/streptomycin	Gibco	Catalog #15140122
Poly-l-lysine hydrobromide	Sigma-Aldrich	Catalog #P2636
Prolong Diamond antifade mountant	Thermo Fisher Scientific	Catalog #P3670
Rapalog	Takara	Catalog #635057
TEMED	Bio-Rad	Catalog #610801

### Electroporation and transfection

For imaging of DIV (day in vitro) 1 to 3 neurons, electroporation was performed, whereas for imaging of neurons >DIV3, Lipofectamine transfection was used. Electroporation of hippocampal neurons was performed directly after dissociation. The dissociated neuron suspension was spun down at 200*g* for 5 min, the supernatant was removed, and neurons were resuspended carefully in electroporation buffer [12.5 mM NaCl, 123 mM KCl, 20 mM KOH, 10 mM EGTA, 4.5 mM MgCl_2_, and 20 mM Pipes (pH 7.2)] mixed with 20% fetal calf serum (FCS). The electroporation buffer and FCS aliquots were stored at −20°C and mixed after being thawed separately. Vigorous resuspension using a pipette can damage the neurons, and small clumps of neurons that did not resuspend after four to five times mixing with the pipette were removed from the solution whenever possible. The resuspended neurons were mixed with 1 to 3 μg of plasmid DNA and placed in electroporation cuvette (density of 200,000 to 1 million neurons per cuvette) (Bio-Rad, GenePulser; 0.2-cm gap) and electroporated in a Nucleofector 2b device (Lonza) on program O-003/rat hippocampal neurons. After electroporation, neurons were diluted in full medium and divided over multiple wells at ~50,000 neurons per 18-mm coverslip, which were coated as described above. For DIV1 neuron imaging, the following amounts of DNA were used: K353/K560 (1.5 to 2 μg), TRIM46 (0.8 μg), K560Rigor (0.3 μg), iLID-CAAX (1 μg), PA-tubulin (2 μg), mRFP-fill (0.3 μg), and PA-Rac1 (1 μg). For Lipofectamine transfections, 50,000 neurons per well of a 12-well plate were directly plated in full medium onto 18-mm coverslips coated as described above and transfected at the indicated times using Lipofectamine 2000 (Invitrogen). For 1 well of a 12-well plate, 1.8 μg of DNA was mixed with 3.3 μl of Lipofectamine in 200 μl of NB and incubated for 30 min at room temperature. Meanwhile, the conditioned medium, the medium in which neurons were cultured, was transferred to a new plate, and transfection medium (NB medium supplemented with 0.5 mM glutamine) was added. The DNA/Lipofectamine mix was added to the neurons in transfection medium and incubated for 45 min at 37°C and 5% CO_2_. Neurons were rinsed by dipping coverslips in prewarmed NB medium and placed back into conditioned full medium for 1 to 2 days before imaging.

U2OS cells were purchased from the American Type Culture Collection and cultured in Dulbecco’s modified Eagle’s medium containing 10% FCS and penicillin/streptomycin (50 μg/ml) at 37°C and 5% CO_2_. Cells were confirmed to be free of mycoplasma. U2OS cells were plated on 25-mm-diameter coverslips 2 days before transfection. For transfection of one well from a six-well plate with a 25-mm coverslip, 6 μl of FuGENE 6 transfection reagent (Promega) was added to 100 μl of Leibovitz’s L-15 medium followed by addition of 0.3 to 2 μg of DNA. The mix was incubated for 5 min at room temperature and then homogeneously added to the medium in a well.

Developmental stages of neurons were categorized as stage 2, stage 3, and stage 4 by morphology (*4*). DIVs were counted after seeding the dissociated neurons: DIV1 to DIV2 (26 to 48 hours), DIV2 to DIV3 (48 to 72 hours), and DIV7 to DIV8 (168 to 192 hours) neurons were used for stage 2, stage 3, and stage 4 of neuron development, respectively (*4*, *12*).

### Live imaging assays

For live-cell imaging, 18-mm coverslips were mounted in a Ludin chamber (Life Imaging Services) covered with 1 ml of conditioned medium. The chamber was mounted in a Tokai hit chamber, maintained at 37°C, and supplied with 5% CO_2_. Cells were imaged using a Nikon spinning disk confocal microscope with the following configuration: Nikon Eclipse Ti (Nikon) with Perfect focus system (Nikon) equipped with Photometrics prime backside illuminated sensor (BSI) camera or Evolve Delta 512 electron multiplying charge-coupled devices (EMCCD) camera (Photometrics); Plan Apo 60× numerical aperture (NA) 1.4 oil or Plan Fluor 40× NA 1.30 oil objectives (both Nikon); Vortran Stradus 405-nm (100 mW), Cobolt Calypso 491-nm (100 mW), and Cobolt Jive 561-nm (100 mW) laser; ASI motorized stage MS-2000-XYZ with piezo top plate; ET-mCherry (49008), ET-GFP (49002), and ET-GFPmCherry (59022) filters (all Chroma); ILAS2 FRAP module (Roper Scientific, now Gataca Systems); spinning disk–based confocal scanner unit (CSU-X1-A1, Yokogawa); and MetaMorph 7.7 software (Molecular Devices).

The electroporation protocol caused death of a small proportion of neurons within 12 to 24 hours after electroporation. Hence, neuron morphology was checked with transmitted light before live-cell imaging. Healthy neurons with low to moderate expression level of protein were carefully chosen for live-cell imaging to avoid overexpression artifacts and phototoxicity. To further reduce phototoxicity, low laser powers corresponding to ~80 W/cm^2^ were used. Exposure time between 200 and 300 ms and an Electron-multiplying (EM) gain of 900 to 950 were used with an EMCCD camera. For Figs. 1 (A, C, and D) and 2B and figs. S1 (A and D), S2B, S3 (A and E), S5B, and S6, images were acquired with 5-min intervals; for Fig. 1E and figs. S2A and S7, images were acquired with 1-min intervals; and for Fig. 1G, images were acquired with 30-s intervals.

### Photoactivation of Rac1 and PA-tubulin

For Rac1 photoactivation, neurons were electroporated with PA-Rac1 construct and human Kinesin-1 [Kif5b(1–353)-GCN4-mcherry]. DIV1 neurons expressing low levels of Kinesin-1 were selected for imaging, and a region of interest (ROI) was drawn around a neurite. Neurons were imaged with 561 nm in the “Dark,” meaning without 405 nm for 15 to 40 min before photoactivation. Using a custom-made journal in MetaMorph software, the 405-nm laser was used to illuminate the ROI every 5 to 10 s for a total of 30 to 40 min. Kinesin-1 was imaged at a rate of 1 frame/min. For the PA-tubulin experiments, neurons were electroporated with PA-tubulin and mCherry-fill constructs and imaged at stage 2 or stage 3. For imaging of stage 4 neurons, neurons were transfected with Lipofectamine with the same constructs and imaged 24 to 48 hours after transfection. Using the ILAS2 FRAP module, single or multiple ROIs of 10 to 15 μm length were drawn along stage 2 neurites (for stage 2 neurons) or axons and dendrites (stage 3 and 4 neurons). All the ROIs on a neuron were illuminated at once with 20 to 27% laser power (0.1 to 1 W/cm^2^) of 405-nm Cobolt Calypso (100 mW) laser. The illumination settings were optimized to achieve the highest green fluorescent protein (GFP) signal in an ROI. Upon photoactivation, the PA-tubulin signal was imaged using 491-nm laser excitation for 30 to 60 min at a rate of 1 frame/min.

### Microtubule anchoring assays

For rapalog-induced microtubule anchoring, neurons were electroporated with FKBP-mCherry-CAAX as a membrane anchor, K560Rigor-HA-FRB as a microtubule anchor, and K353-GCN4-mCherry for monitoring Kinesin-1 transport. DIV1 stage 2 neurons with low expression levels of Kinesin-1 were selected. Rapalog was added at a concentration of 200 nM to anchor the Kinesin-1 rigor-decorated microtubules to the cell membrane. As a control, the experiment was repeated using K560Rigor-HA without the FRB domain (Fig. 2C and fig. S5). Cells were imaged either live or after fixation with prewarmed 4% paraformaldehyde in phosphate-buffered saline (PBS) after 2 hours of rapalog addition. For the long-term microtubule anchoring assay in Fig. 2, 10 nM rapalog was added to stage 2 neurons for 24 to 48 hours.

For light-induced microtubule anchoring, neurons were electroporated with iLID-CAAX as a membrane anchor, K560Rigor-mVenus-SSPB as a microtubule anchor, and K353-GCN4-mCherry for monitoring Kinesin-1 transport. Neurons were imaged on the Nikon spinning disk microscope described above, using the 40× oil objective and the ILAS2 FRAP module for photoactivation. Neurons were protected from stray light before imaging by wrapping the 12-well plate in aluminum foil. The microscope set up was surrounded by black curtains to avoid any stray light activating the photosensitive module. DIV1 stage 2 neurons with low expression level of Kinesin-1 were selected for imaging. Kinesin-1 (Kif5b-mCherry) was imaged with 561-nm laser every 60 s, while local illumination using a 405-nm laser was performed every 5 to 10 s to achieve light-induced anchoring. As a control experiment, neurons were electroporated with iLID-CAAX and K560Rigor-mVenus without the SSPB domain for light-induced microtubule anchoring (Fig. 2J and fig. S5).

### Pharmacological treatments and immunostainings

Neurons were electroporated with a Kinesin-1 construct and plated onto coated 18-mm coverslips. Thirty hours after plating, Rac inhibitor EHT1864 (20 μM), Cdc42 (20 μM) inhibitor, or jasplakinolide (100 nM) was added to the cells for 2 hours and then fixed for immunostaining (Table 3). Dimethyl sulfoxide or distilled water was added as control. DIV1 neurons were fixed in prewarmed (37°C) 4% paraformaldehyde in PBS for 15 min. After three washes with PBS, cells were permeabilized with 0.2% Triton X-100 in PBS for 10 min and then washed three times with PBS. Cells were blocked for 45 to 60 min with 3% bovine serum albumin (BSA) in PBS followed by 1-hour incubation in primary antibodies diluted in 3% BSA in PBS. After three washes with PBS, cells were incubated with secondary antibodies diluted in 3% BSA in PBS for 45 min. After three washes with PBS, coverslips were mounted in ProLong Diamond antifade mountant.

**Table 3.** Table of antibodies.

**Antibody**	**Source**	**Reference**
Goat anti-mouse Alexa Fluor 488	Life Technologies	Catalog #A11029
Goat anti-mouse Alexa Fluor 568	Life Technologies	Catalog #A11031
Goat anti-rabbit Alexa Fluor 488	Life Technologies	Catalog #A11034
Goat anti-rat Alexa Fluor 594	Life Technologies	Catalog #A11007
Mouse anti-Tau	Merck	Catalog #MAB3420
Mouse anti-tubulin acetylated	Sigma-Aldrich	Catalog #T7451
Phalloidin–Alexa Fluor 488	Thermo Fisher Scientific	Catalog #A12379
Rabbit anti-TRIM46	C. Hoogenraad	(*17*)
Rabbit anti-GFP	MBL International	Catalog #598
Rabbit anti-laminin antibody	Abcam	Catalog #ab11575
Rat anti-tubulin antibody [YL1/2]	Abcam	Catalog #ab6160

### Polyacrylamide hydrogel stiffness gradients

The hydrogel stiffness gradients for culturing embryonic rat hippocampal neurons were based on polyacrylamide. We combined elements from other approaches (*26*, *29*, *40*) to optimize the fabrication of hydrogel gradients. First, coverslips were silanized according to a method described previously (*40*). Briefly, 18-mm glass coverslips were plasma-treated using a plasma cleaner (Harrick Plasma Cleaner, PDC-002) for 3 min and immediately treated with 2% (v/v) 3-(trimethoxysilyl)propyl methacrylate and 1% (v/v) acetic acid in ethanol for 10 min in a beaker with occasional manual shaking. Coverslips were washed twice with ethanol and then air-dried using coverslip holders. Dried coverslips were baked at 120°C for 2 hours and stored in a dry place at room temperature for up to a month. To generate polyacrylamide hydrogel gradients, two acrylamide solutions, soft (0.4 kPa) and stiff (7 kPa), were prepared to create stiffness gradient of 0.4 to 7 kPa. Gel premixes were prepared by mixing acrylamide and bis-acrylamide solutions. The desired Young’s modulus of the premixes was adjusted by mixing predefined ratios of 40% (w/v) acrylamide and 2% (w/v) *N*,*N*-methyl-bis-acrylamide cross-linker in PBS. Gel premix solutions (200 μl) corresponding to 0.4 kPa [5% acrylamide (AA) and 0.04% bis-AA] and 7 kPa (12% AA and 0.03% bis-AA) were prepared. Fluorescently labeled 0.2-μm carboxylated beads [dark red fluorescent (660/680)] were added at 3.6 × 10^9^ beads per 100 μl to the stiff premix solution. The bead solution was vortexed for 3 min before addition to the gel solution. Silanized coverslips were placed onto parafilm. Polymerization was initiated by adding 2 μl of 10% ammonium persulfate and 1 μl of *N*,*N*,*N*′,*N*′-tetramethylethylenediamine (TEMED) to the solutions. Eight-microliter droplets of both the stiff and soft premixes were put onto the silanized coverslip, 2 to 3 mm apart on one side of the 18-mm coverslip. A 14-mm glass coverslip was placed onto the droplets by gently dropping it from one end to the other end of the 18-mm coverslip, leading to in situ mixing of acrylamide solution by diffusion (*29*). Gels were polymerized for 45 to 50 min at room temperature and then covered with 2 ml of PBS for 30 min to facilitate detachment of gels from coverslips. Then, the 14-mm coverslip was gently detached using needle and tweezers. Acrylamide hydrogels attached strongly to the silanized coverslips and were placed in PBS solution at 4°C up to 4 to 5 days. To allow laminin to adhere to the hydrogel substrates, gels were incubated in hydrazine hydrate for 4 hours and 5% acetic acid for 1 hour and thoroughly washed with PBS three times. Gels were incubated in poly-l-lysine (37.5 μg/ml) in PBS for 2 days and then incubated with laminin (12.5 μg/ml) in PBS for 2 hours before plating the neurons. To measure the coating of laminin on hydrogel gradient substrates, gels were incubated with rabbit anti-laminin antibody diluted 1:200 in 3% BSA in PBS. Gels were washed three times with PBS and then incubated with anti-rabbit Alexa Fluor 488 antibody for 1 hour followed by three washes with PBS. Gels were mounted onto glass slides using ProLong diamond mountant. Images of beads and laminin were acquired every 300 μm across the gradient using 40× objective of Nikon spinning disk microscope.

A control experiment was performed to test whether axon formation is sensitive to a gradient of fluorescent beads (fig. S9). Rigidity (3 kPa) was chosen as an intermediate rigidity of the 0.4- to 7-kPa gradient for the control experiment. Two polyacrylamide premixes corresponding to 3 kPa (7.5% AA and 0.06% bis-AA) were prepared. Fluorescent beads were added to one solution, and the gradient was prepared as described above. Bead gradient was analyzed, and axon orientation was determined as described below in image analysis section.

### Rheology measurements

Mechanical properties of the hydrogels were assessed by oscillatory shear measurements, using a DHR-2 rheometer (TA Instruments), with a plate-plate geometry (20 mm diameter) and Peltier hood to prevent evaporation. The viscoelastic linear regime (LVR) was determined for all formulations by an amplitude sweep test at angular frequency ω = 10 rad/s. Hydrogel formation was monitored by a time sweep test (oscillation strain γ = 2%, ω = 10 rad/s, 30 to 40 min), and frequency-dependent measurements were carried out in the frequency range 0.1 to 100 rad/s, at a strain of γ = 2% (safely within LVR). All measurements were conducted at 20°C and in triplicates, unless noted otherwise. The stiffness of the gels was taken as the value of the storage modulus *G*′(ω) at ω = 1 rad/s, as measured by the frequency sweep test. The values are reported in fig. S8.

### Analysis of stiffness gradients

Images of neurons on gradient gels with fluorescent beads were imaged using a 40× objective on the spinning disk microscope described above. Single, isolated neurons were imaged within 200 μm × 150 μm regions by acquiring a *z*-stack with 1-μm *z*-plane distance covering 12- to 15-μm distance. To analyze the rigidity along the growing neurites or axon, multichannel images comprising the bead channel and Kinesin-1 or Tau staining were acquired. A maximum intensity *z* projection was generated for all channels using Fiji. Beads were counted using the <Find maxima> plugin in Fiji within a 300 pixel × 300 pixel (~45 μm × 45 μm) grid area of the bead image. The value of prominence in the plugin was carefully adjusted for each experiment to obtain detection of all beads in the ROI and to avoid false-positive and false-negative detection of beads.

To derive the relation between the number of beads and local gel stiffness, a gel consisting of only the stiffer, bead-containing solution was made. Images of the beads were taken in a similar fashion as described above, resulting in an estimate for the number of beads per 10,000 μm^2^ for the stiffest rigidity of 7 kPa, whereas the softer gel (0.4 kPa) featured density of zero beads per 10,000 μm^2^. On the basis of previous work (*29*), a linear relationship between the number of beads and stiffness within 0.4- to 7-kPa rigidity range was assumed, resulting in the following calibration scale: *y* = 0.44 + 0.001987*x, where *y* is the stiffness in kPa and *x* is the number of beads per 10,000 μm^2^.

Because beads were counted in grid areas of ~45 μm × 45 μm, an interpolation method was used to better estimate local gradients. The interpolation method (image resize option, bilinear interpolation) in Fiji performs interpolation but places the original pixel values in the top left of each interpolated region. To correct for this shift, we performed “Matrix padding” and then cropped the image so that the value of the bead count was at the center of each grid unit of 45 μm × 45 μm in the interpolated image (fig. S8). Only ROIs that featured uniform bead gradients were chosen for analysis. Although the global gradient of the gels was known, for each analyzed neuron, we determined the exact local direction of the gradient. To this end, an 80-μm-radius circle was drawn centered around the cell body and the maximum difference in stiffness was determined for 18 equidistant pairs of points on these circles. The points corresponding to the maximum difference defined the axis against which the angles of axons, dendrites, and immature neurites were measured. Neurites with angles within the range of <90° and >−90° were counted as facing toward the softer matrix, whereas neurites with angles within the range of >90° and <−90° are counted as facing toward the stiffer matrix.

### Analysis of live imaging assays

Time-lapse TIFF images were processed in Fiji. Intensities of the fluorescently labeled proteins were measured by drawing ROIs enclosing different neurites or the soma. Background subtraction was performed by subtracting the mean background intensity multiplied by the ROI area from the raw intensity of the ROI for every frame. In some cases where ROI intensities were very low, the mean value of the background intensity was subtracted to avoid negative intensity values. For generating movies, the average background intensity was subtracted from the entire stack of images and a Gaussian blur of one pixel was applied. For the TRIM46 intensity analysis in fig. S1, the TRIM46 intensity in each neurite was measured at every time frame for a total of 2 hours. Then, the TRIM46 intensity at each frame was divided by the minimum TRIM46 intensity in a neurite during those 2 hours to obtain the fold change of intensity at every frame, and the maximum fold change for each neurite was plotted. For quantification of Kinesin-1 intensity, the intensity in each neurite was measured at every time frame and divided by the total Kinesin-1 intensity within all neurites to obtain the percentage of Kinesin-1 accumulation in a neurite at every time frame.

To analyze microtubule network movement using PA-tubulin, the time-lapse movies of each photoactivated region along axon, dendrites, or stage 2 neurites were converted into a kymograph. To generate a kymograph, a segmented line of 3.5 μm thickness, starting closer to the cell body, was drawn along the photoactivated GFP tubulin regions. The “KymoResliceWide” plugin in Fiji was used to obtain the kymographs. Negative and positive velocities were assigned to the retrograde (toward the cell body) and anterograde (away from the cell body) movements, respectively.

### Statistical analysis

Datasets were tested for normal (Gaussian) distribution using the Shapiro-Wilk normality test. In case of normal distribution, statistical comparisons between two groups were done using two-tailed Student’s *t* test. If data did not follow a normal distribution, a Mann-Whitney nonparametric test was used to compare the differences between different conditions. To compare proportions of different outcomes between two or more conditions, the chi-square test was used. To compare difference between values before and after treatment for the same sample, the nonparametric Wilcoxon matched pair test was used. Analysis of variance (ANOVA) was used to compare the differences between more than two groups. Data were plotted, and statistical tests were performed using GraphPad Prism 9.
