# Supplementary material for: Local changes in microtubule network mobility instruct neuronal polarization and axon specification
Source: Sci Adv. 2022 Nov 4;8(44):eabo2343. doi: 10.1126/sciadv.abo2343 (PMC9635826; doi:10.1126/sciadv.abo2343)
Supplement: Supplementary file 1 — Figs. S1 to S10 [file sciadv.abo2343_sm.pdf]

Science Advances
AAAS

Supplementary Materials for

Local changes in microtubule network mobility instruct neuronal polarization and axon specification

Mithila Burute *et al.*

Corresponding author: Lukas C. Kapitein, l.kapitein@uu.nl

Sci. Adv. **8**, eabo2343 (2022)
DOI: 10.1126/sciadv.abo2343

The PDF file includes:

Figs. S1 to S10
Legends for movies S1 to S12

Other Supplementary Material for this manuscript includes the following:

Movies S1 to S12

SUPPLEMENTAL FIGURES

Figure S1. Microtubule network flow and TRIM46 mobility reduce during neuron development

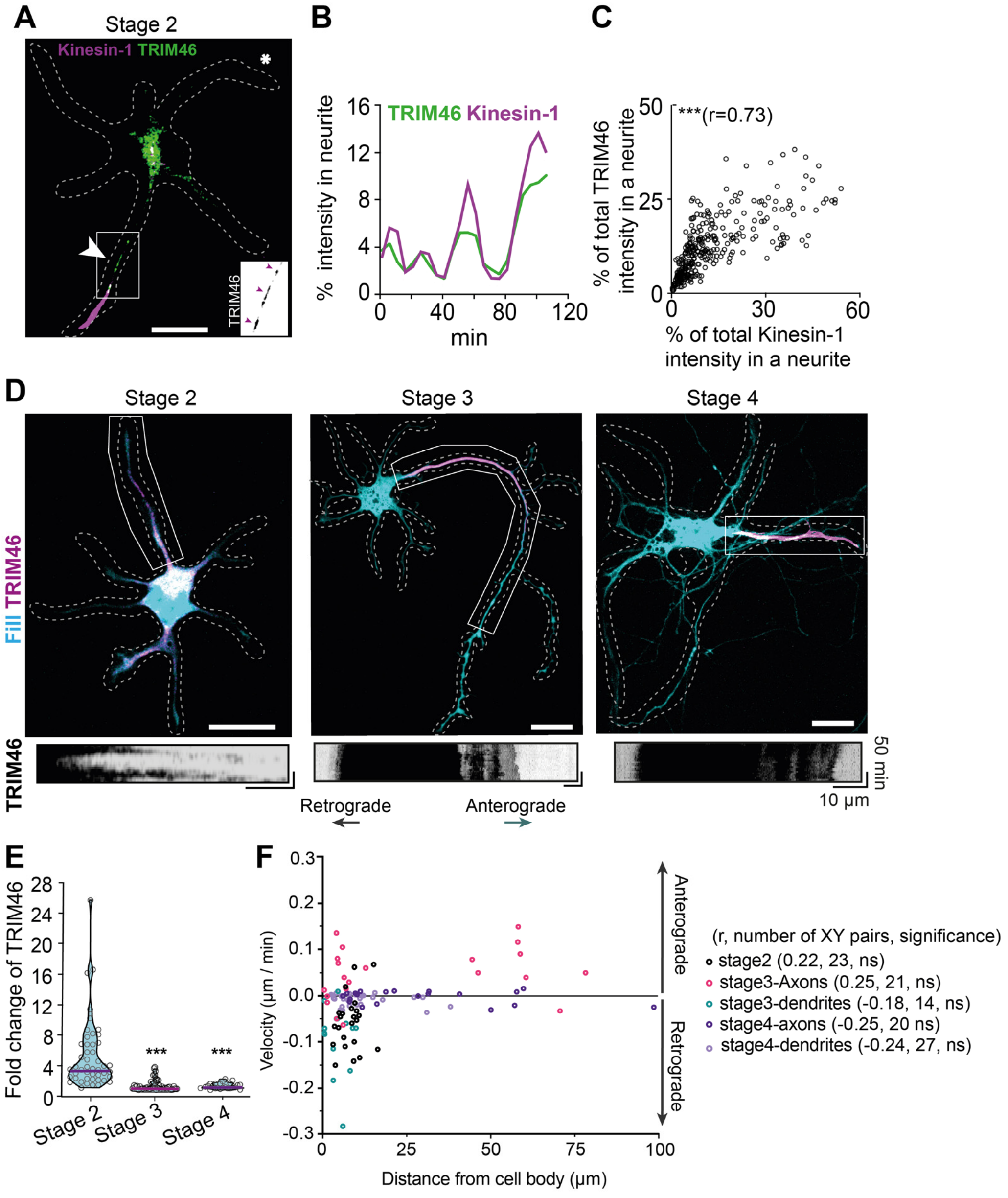

(A) Appearance of TRIM46 (green) in a neurite (white arrowhead) is associated with selective transport of Kinesin-1(magenta). Inverted grey inset shows TRIM46 patches (magenta arrowheads) in the neurite. Scale bar, 10 µm.

(B) Change in percentage of intensity of TRIM46 and Kinesin-1 in the neurite marked with * in (A).

(C) Correlation between percentage of total TRIM46 and Kinesin-1 intensity in a neurite at various time points (n=322 *xy* pairs, 20 neurites from 4 neurons). Grey shades represent data from different neurons. *** $p \leq 0.001$. r is Pearson correlation coefficient (Pearson correlation test).

(D) Snapshots of stage 2-4 neurons expressing TRIM46 (magenta) and fill (cyan). Bottom: inverted grey scale images show kymographs of TRIM46 within the neurites marked by white boxes on top images Scale bar, 20 µm.

(E) Maximum fold change of TRIM46 intensity in a neurite within 2 h. Stage 3 and 4 values were statistically compared with stage 2. ***$P \leq 0.001$ (Mann-Whitney test).

(F) Velocity of PA-tubulin regions plotted against their distance from the cell body. Pearson correlation coefficient (r), number of XY pairs and significance of statistical test are reported. ns $P > 0.05$ (Pearson correlation test).

Figure S2. Microtubule retrograde flow precedes Kinesin-1 exit in stage 2 neurons

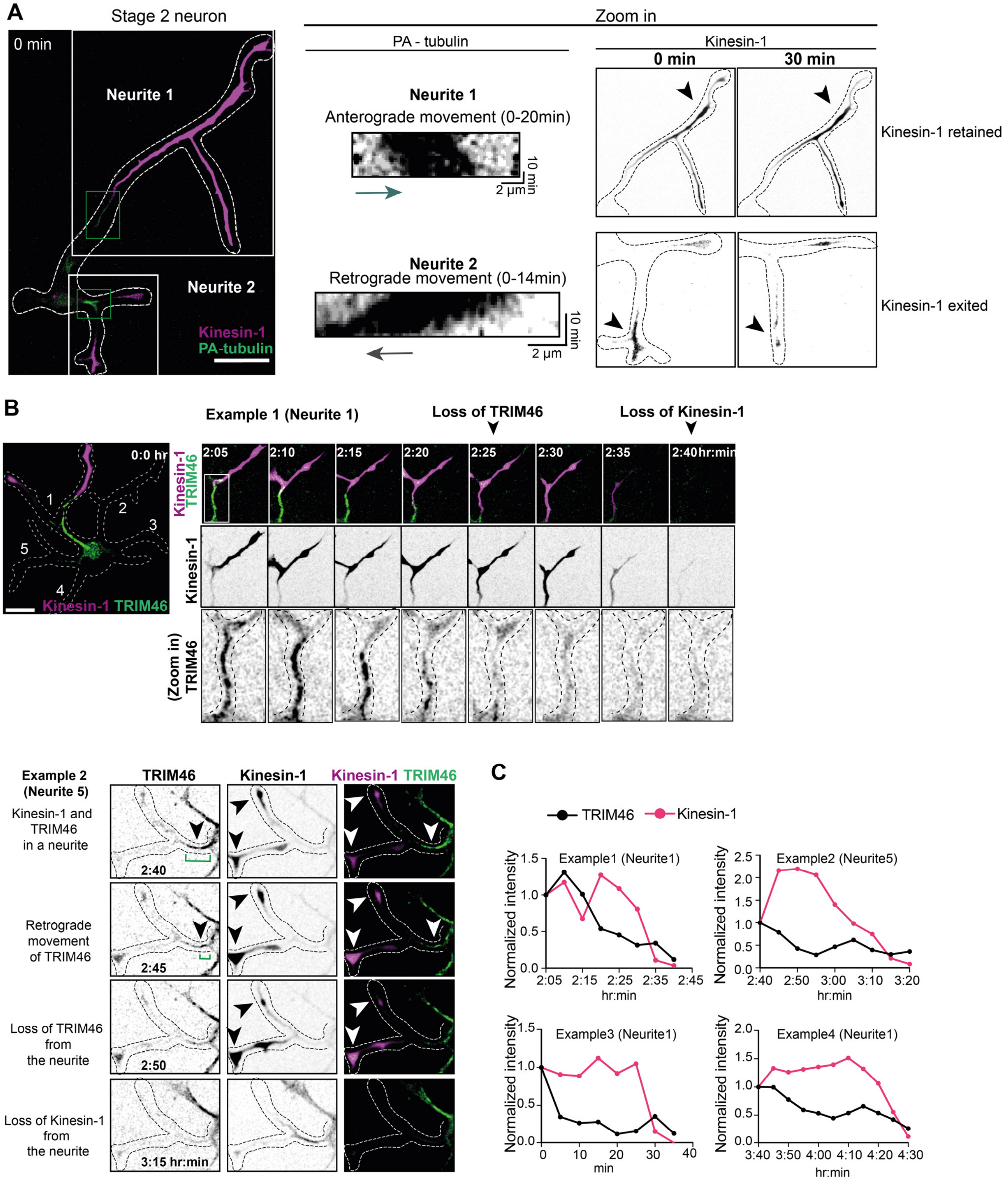

(A) Stage 2 neuron showing changes in Kinesin-1 accumulation associated with PA-tubulin movement. Boxes in left image show region of PA-tubulin used for kymographs on the right. Neurite 1 has anterograde microtubule network flow and retains Kinesin-1 during 30 min. Neurite 2 has retrograde network flow and loses Kinesin-1 within 30 min. Left image: Scale bar, 20 µm.

(B) Example of stage 2 neuron from Fig1D and Movie 4 showing that TRIM46 exit precedes Kinesin-1 exit. Scale bar, 10 µm.

(C) Traces of Kinesin-1 and TRIM46 normalized intensity in different neurites of stage 2 neuron from Fig1D.Time points are indicated as in Movie 4. TRIM46 intensity is measured at the base of the neurite, whereas Kinesin-1 intensity is measured in the entire neurite.

Figure S3. Validation of the microtubule anchoring assay

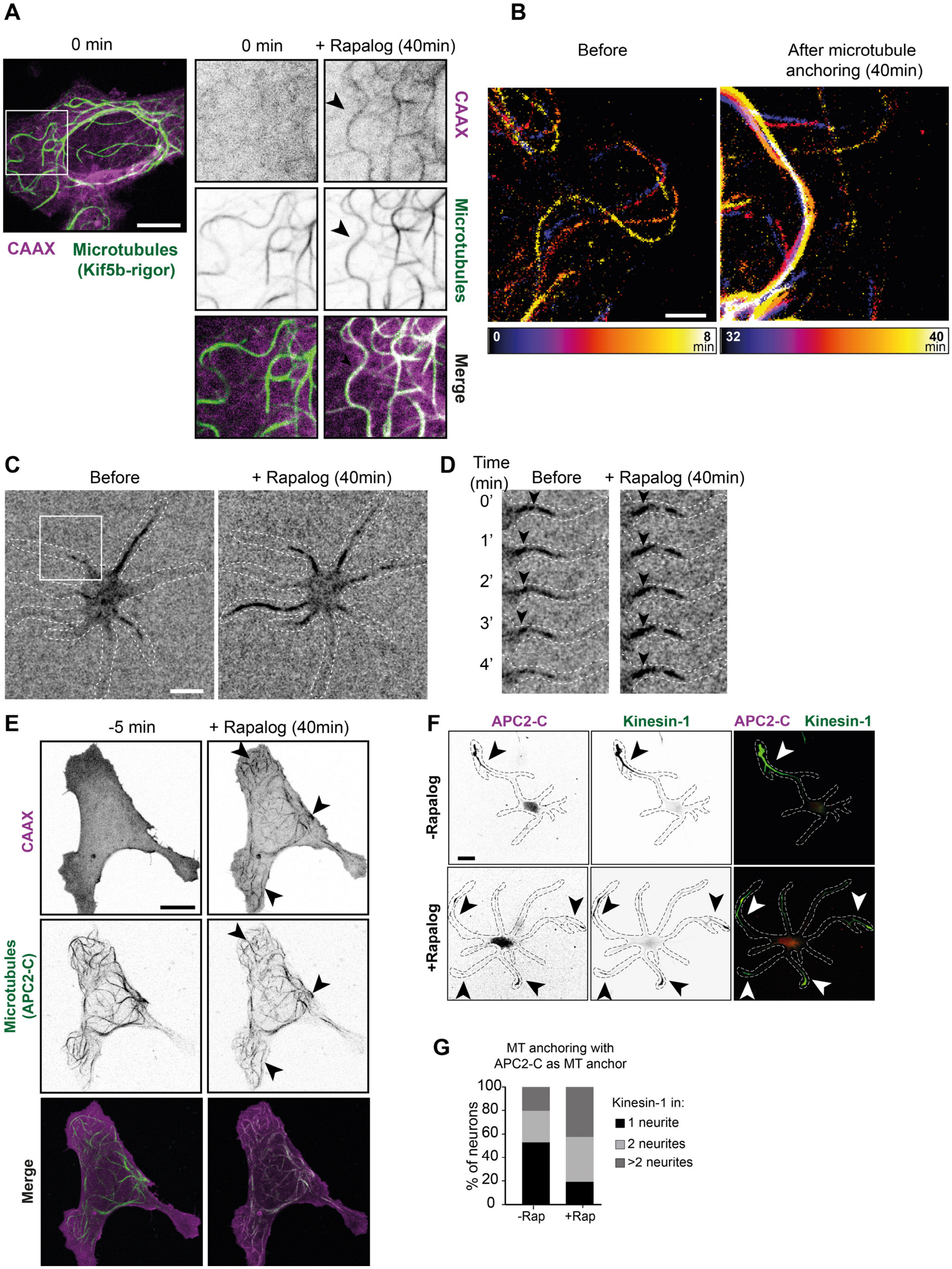

(A) Left: U2OS cell expressing CAAX-FKBP and microtubules decorated with the FRB-microtubule anchor (Kif5b-rigor) before Rapalog addition (time 0 min). Right: zooms of the area in rectangle on left image. Individual images of CAAX, FRB-microtubule anchor, and merge, before (0 min) and after Rapalog addition (40 min). Black arrowheads in the CAAX channel show appearance of microtubule morphology after Rapalog addition. Scale bar, 10 µm.

(B) Temporal color-coded overlays from a time-lapse recording demonstrating reduced microtubule movement after Rapalog addition. Scale bar, 2 µm.

(C) Images of TRIM46 in a Stage 2 neuron before and after Rapalog addition. Scale bar, 10 µm.

(D) Time series of the neurite indicated in the box in (C). Before Rapalog, the TRIM46 patch is lost from the neurite whereas after Rapalog addition, the TRIM46 patches are immobile.

(E) U2OS cell expressing CAAX-FKBP and microtubules decorated with the FRB-microtubule anchor (APC2-C). Individual images of CAAX, FRB-APC2-C, and merge, before (-5 min) and after Rapalog addition (40 min). Black arrowheads in the CAAX channel show appearance of microtubule morphology corresponding to the microtubules channel after Rapalog addition. Scale bar, 10 µm.

(F) Stage 2 neuron expressing CAAX-FKBP and microtubules decorated with the FRB-microtubule anchor (APC2-C) and immunostained for Kinesin-1. Without Rapalog, Kinesin-1 accumulated in a single neurite whereas upon microtubule anchoring by Rapalog addition, Kinesin-1 accumulated in multiple neurites. Scale bar, 10 µm.

(G) Percentage of stage 2 neurons with Kinesin-1 accumulation in 1, 2 or >2 neurites. n=51 neurons without Rapalog (-Rap) and n=73 neurons with Rapalog (+Rap).

Figure S4. Local anchoring of microtubules drives selective TRIM46 accumulation into a neurite

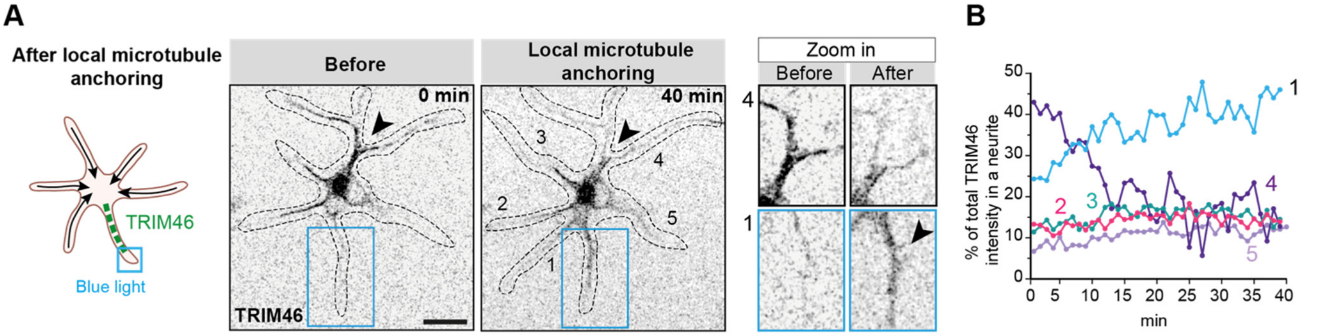

(A) Snapshots of a stage 2 neuron expressing iLID-CAAX, SSPB-microtubule anchor and TRIM46. Upon blue light illumination, TRIM46 is relocated from neurite 4 at 0 min to neurite 1 at 40 min. Blue rectangle represents area illuminated with blue light. Black arrow marks area with reduction of TRIM46 intensity from neurite 4 upon microtubule anchoring. On the right, zoomed-in images of neurite 1 and 4 showing loss of TRIM46 patches from neurite 4 and increase in TRIM46 intensity in neurite 1, marked by arrowhead. Scale bar, 10 µm.

(B) Quantification of percentage of total TRIM46 intensity in neurites after microtubule anchoring. Blue trace represents the neurite that was illuminated with blue light (neurite 1).

Figure S5. Control experiments for the global and local microtubule anchoring assays

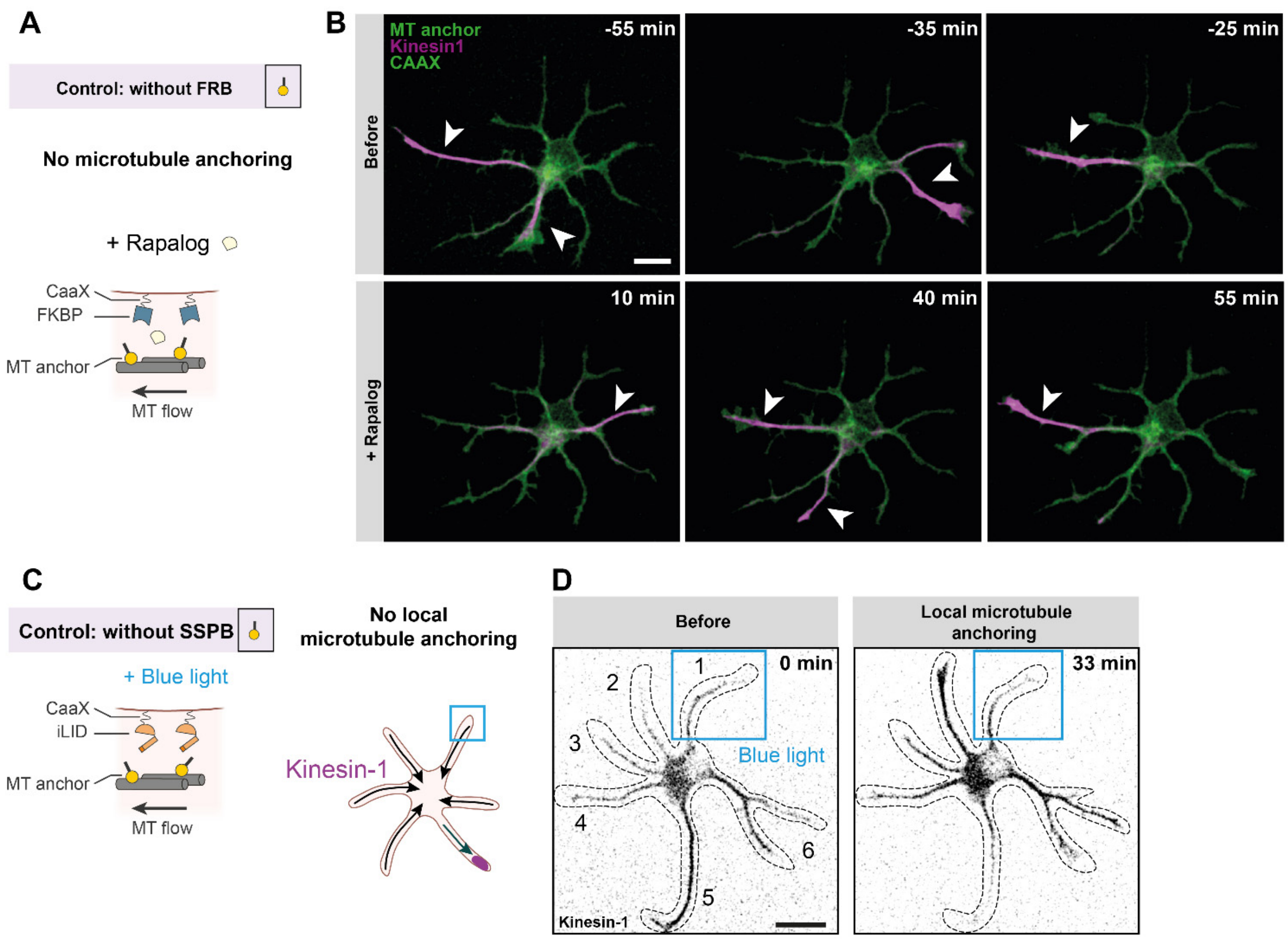

(A) Control experiment for Rapalog-induced global microtubule anchoring assay. Rapalog addition cannot induce dimerization of FKBP-CAAX to microtubule anchor without the FRB domain. Consequently, microtubules do not get anchored to the cell membrane after Rapalog addition.

(B) Stage 2 neuron expressing Kinesin-1(magenta), FKBP-CAAX (green) and MT-anchor without FRB (green). Kinesin-1 fluctuations between neurites continue after Rapalog addition. Scale bar, 10 µm.

(C) Control experiment for light-induced local microtubule anchoring assay. Blue light illumination cannot induce binding of iLID-CAAX to microtubule anchor without the SSPB domain. Consequently, microtubules do not get anchored to the cell membrane after blue light illumination.

(D) Images of stage 2 neuron expressing iLID-CAAX, microtubule anchor without SSPB and Kinesin-1(grey). Blue rectangle represents area that was illuminated with blue light. Kinesin-1 accumulation changes from neurites 5 and 6 to 2 and 6 after microtubule anchoring but does not enrich into neurite 1, which was illuminated with blue light. Quantification of multiple neurons is in Fig. 2J.

Figure S6. Kinesin-1 fluctuations stop upon Rac1 inhibition in stage 2 neuron

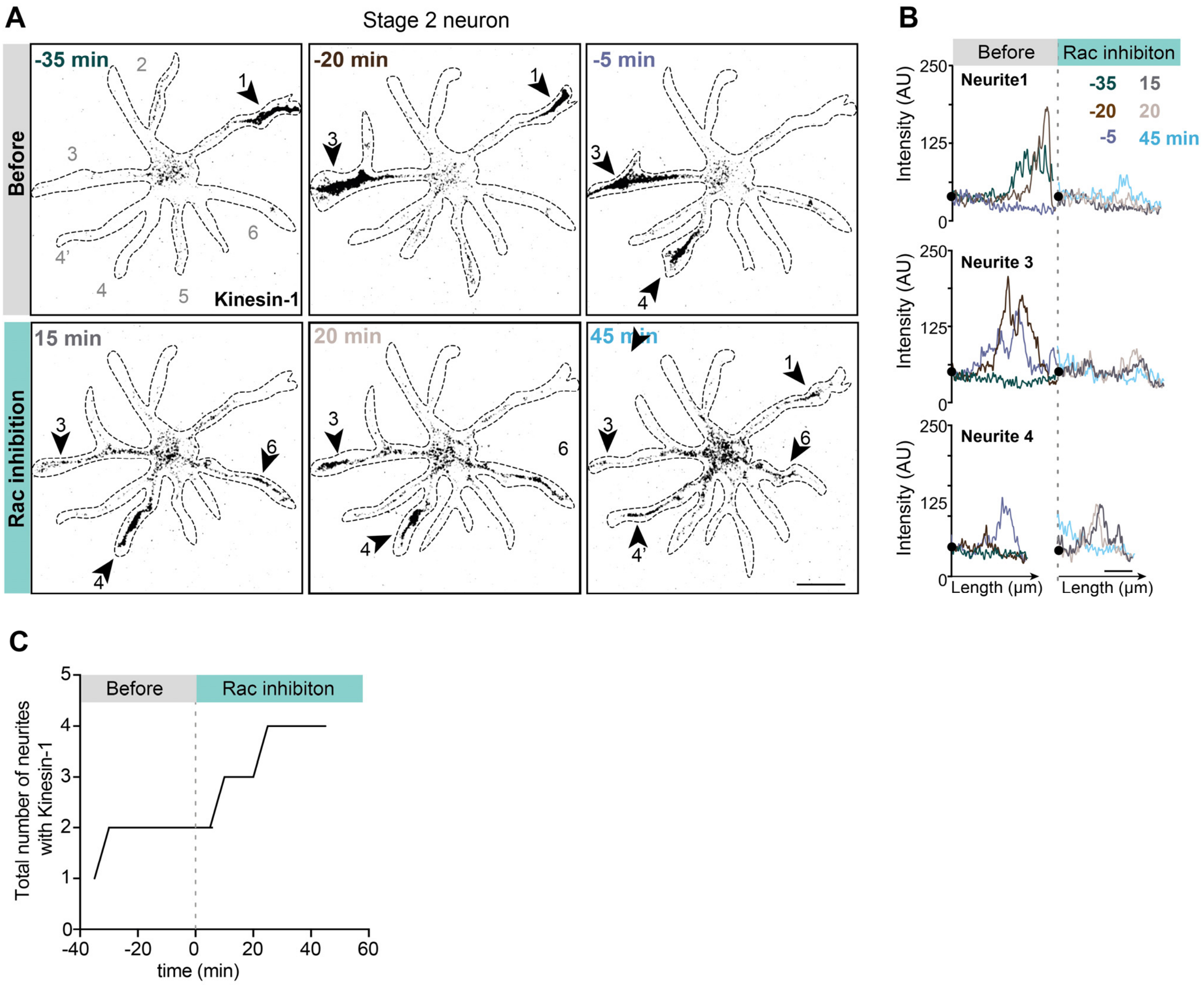

(A) Selected frames from a time-lapse movie of a stage 2 neuron expressing Kinesin-1. Top three frames show change of selective accumulation of Kinesin-1 from neurite 1 (-35’) to neurite 3 (-20’) and to neurite 3 and 4 (-5’). The (transient) selective accumulation of Kinesin-1 is lost upon inhibition of Rac1 resulting in its sustained accumulation in neurites 1, 3, 4 and 6. Scale bar, 10 µm.

(B) Intensity profiles along the length of neurites 1, 3 and 4 for 6 frames shown in (A). Colors are assigned to different time frames. Black dot represents the position of cell body. AU., arbitrary units.

The Rac inhibitor was added at time point 0’ which is indicated by the dotted line. Scale bar, 10 µm.

(C) Graph showing that the total number of neurites with a Kinesin-1 accumulation increases upon Rac inhibition in the neuron in (A).

Figure S7. Local Rac1 activation induces anterograde movement of the microtubule network in stage 2 neuron

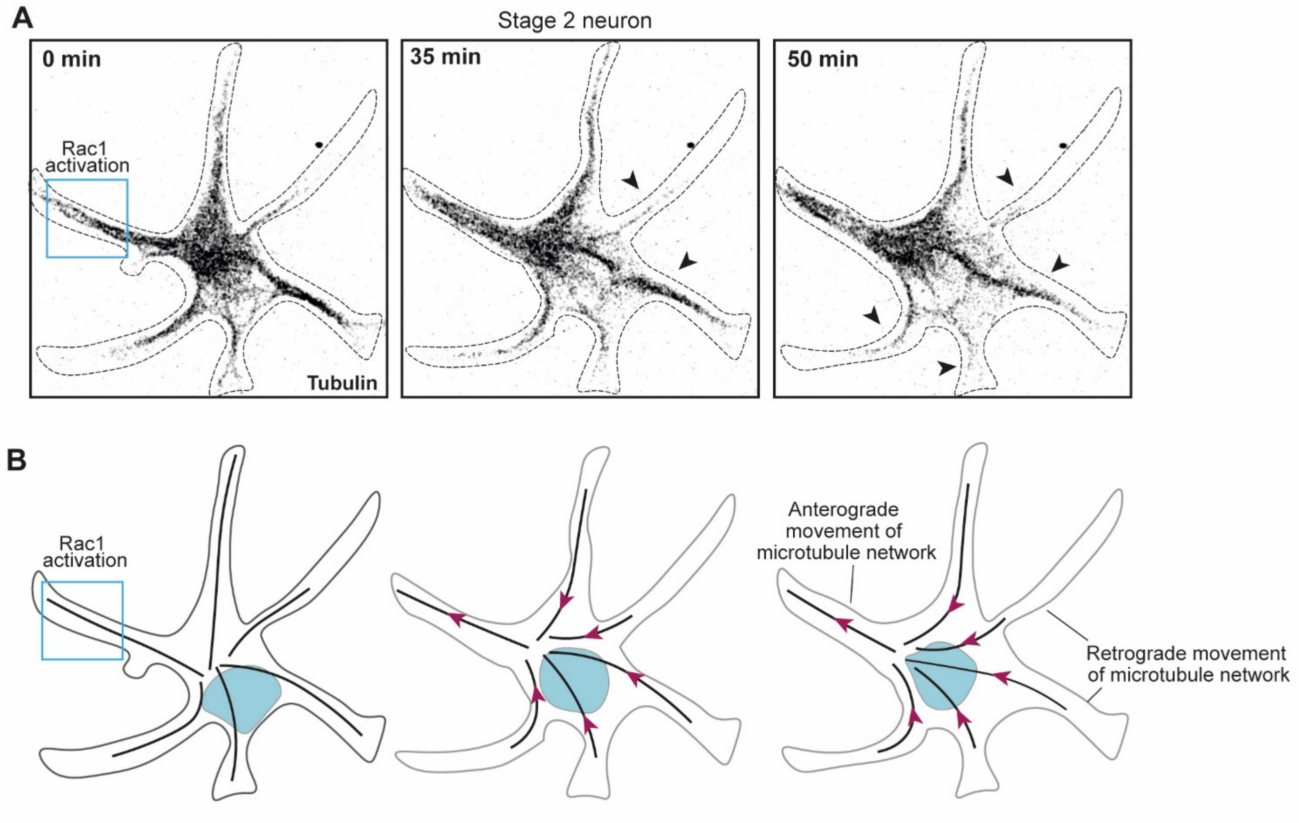

(A) Local activation of Rac1 in a neurite of a stage 2 neuron expressing alpha-tubulin (grey). Black arrowheads indicate reduction of microtubule network intensity from neurites compared to first frame.
(B) Illustration of the neuron in (A) showing anterograde movement of microtubules in the Rac1 activated neurite, while retrograde movement of the microtubule network is seen in non-activated neurites.

Figure S8. Fabrication of stiffness gradients gels and workflow of image analysis

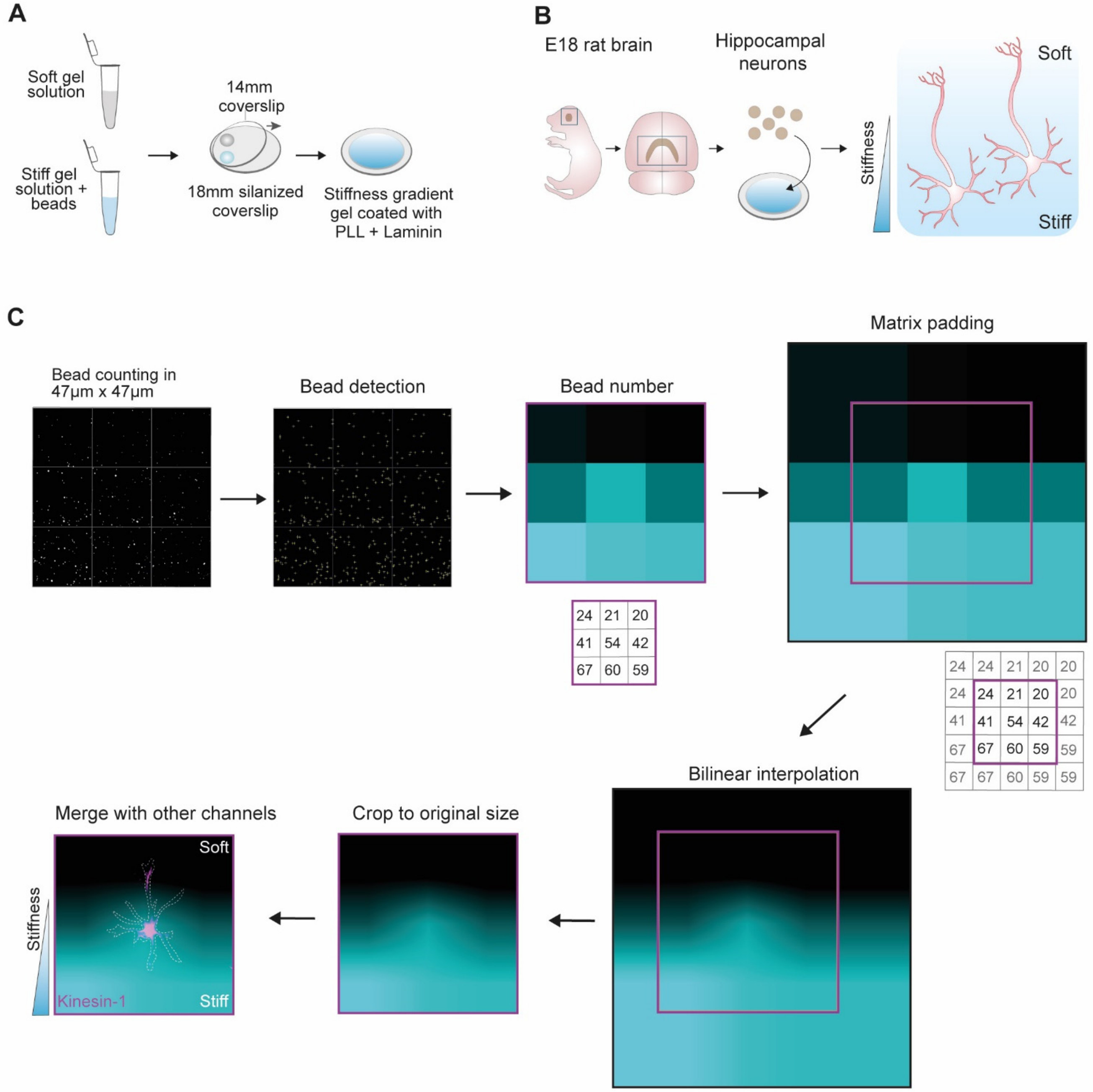

D

Sample (kPa)	Shear modulus (kPa) at 1 rad/s (20°C)
0.4	0.44 ± 0.02
3	3.26 ± 0.08
7	6.87 ± 0.4

(A) Pre-mixes of gel solutions for stiff and soft gels were prepared and from each solution one drop of 8 μl was placed onto a silanized glass coverslips. A 14 mm glass coverslip was placed onto the droplets by gently dropping it from one end to the other end, followed by polymerization. After polymerization, gels were coated with poly-L-lysine (PLL) and Laminin.

(B) Hippocampal neurons from embryonic rat brain (E18 stage) were dissociated and seeded onto the PLL and Laminin coated gradient hydrogels. Neuron growth was tracked on different days to capture different stages of development.

(C) The stiffness gradient of acrylamide hydrogels was derived from images of fluorescent beads embedded within the gel. Beads were detected in squares of fixed size. In this example, a 3x3 pixel image was generated using the bead counts within the 9 squares. Before interpolation, extra rows and columns were added at the edges of the matrix (numbers in grey, see methods), generating a 5x5 matrix. This 5x5 pixel image was scaled up using bilinear interpolation, followed by cropping to the original size of the image (magenta box). The cropping area was positioned so that the original value of the bead count in the grid was at the center of each grid area in the interpolated image. The final cropped gradient image was merged with other imaging channels.

(D) Shear modulus values of gel premixes of different stiffnesses. The premixes for 3 and 7 kPa gels contained beads.

Figure S9. Control experiment showing bead gradients do not affect axon formation

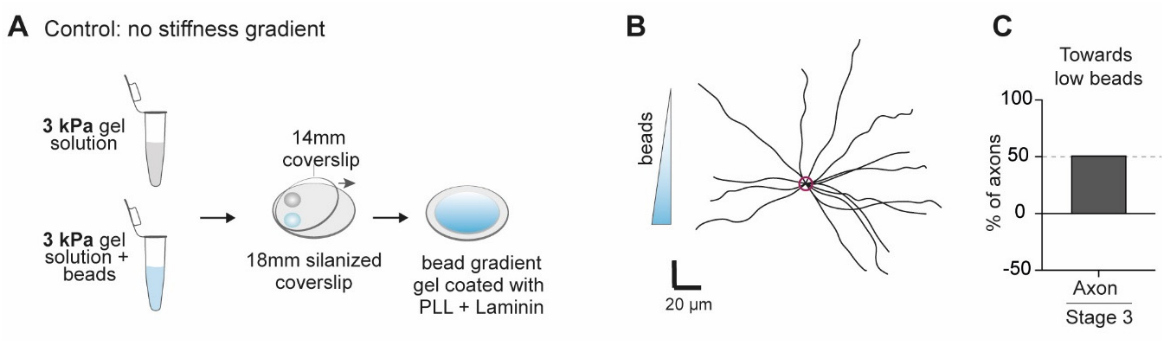

(A) A bead gradient was generated by mixing a solution for 3 kPa containing fluorescent beads with a similar solution without beads.

(B) Traces of stage 3 axons on 3 kPa polyacrylamide gels with a bead gradient show no bias for the direction of the bead gradient. Scale bar 20 µm (*x,y*) .

(C) Quantification of percentage of axons growing towards lower bead density (n=14).

Figure S10. Measurement of stiffness gradient along axons and neurites

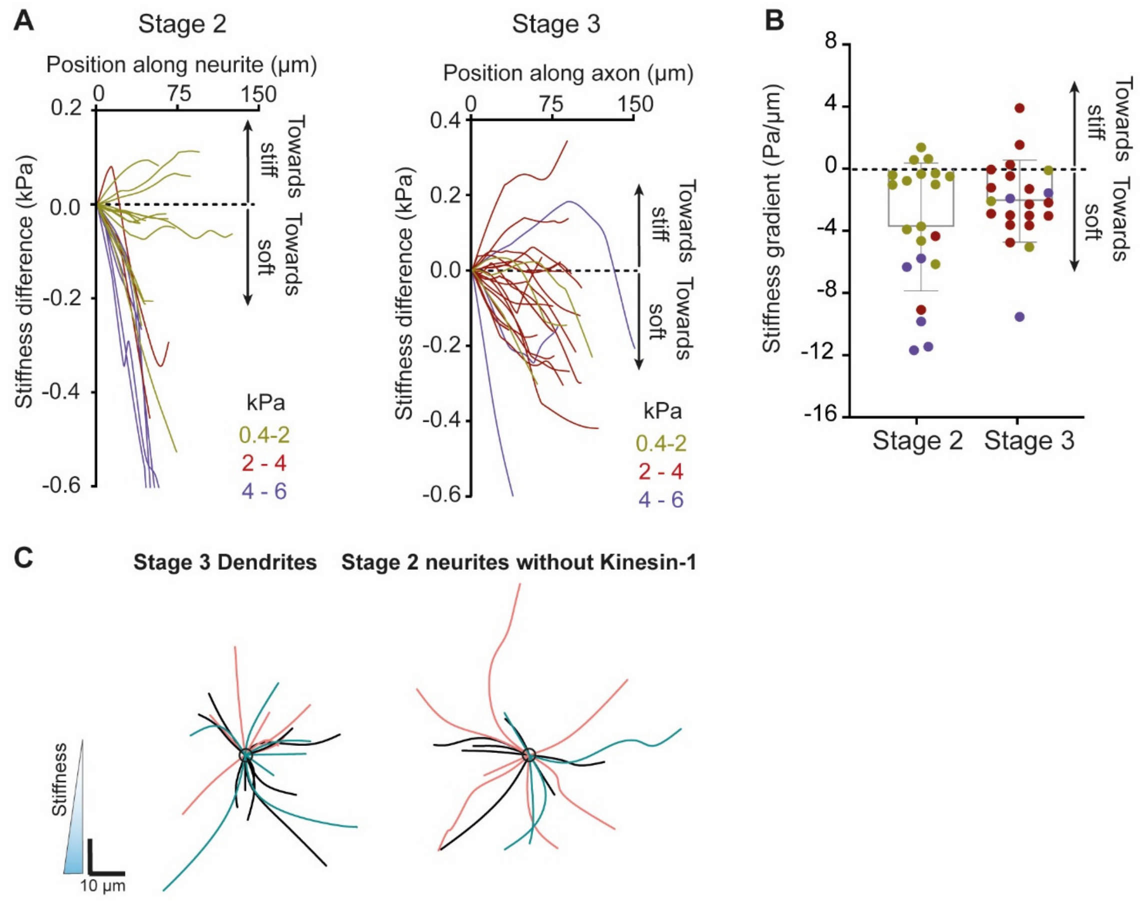

(A) Stiffness difference relative to the cell body, along the length of stage 3 axons (n=22) and stage 2 neurites with Kinesin-1 (n=21). Colors represent different rigidity ranges for the matrix near the cell body.

(B) Average stiffness gradient for the traces shown in (A). Error bars represent S.D.

(C) Orientations of dendrites (n=25) and stage 2 neurites (n=18) on elasticity gradients from 3 independent experiments. Different colors represent traces from independent experiments.

SUPPLEMENTARY MOVIES

Supplementary Movie 1. Kinesin-1 fluctuations between neurites of stage 2 neuron. Scale bar 10 µm. This video complements Fig. 1.

Supplementary Movie 2. TRIM46 fluctuations between neurites of stage 2 neuron. Scale bar 10 µm. This video complements Fig. 1.

Supplementary Movie 3. Anterograde and retrograde movement of TRIM46 patches in stage 2 neurons. Scale bar 5 µm. This video complements Fig. 1.

Supplementary Movie 4. Selective transport of Kinesin-1 is associated with TRIM46 accumulation in the neurites. Arrowheads indicate retrograde movement of TRIM46. Scale bar 10 µm. This video complements Fig. 1.

Supplementary Movie 5. Microtubule network mobility at different stages of neuron development. This video complements Fig. 1.

Supplementary Movie 6. Changes of TRIM46 accumulation in neurites reduce during neuron development. This video complements Fig. S1.

Supplementary Movie 7. Retrograde microtubule flow precedes Kinesin-1 exit. This video complements Fig. S2.

Supplementary Movie 8. Appearance of microtubules near the plasma membrane upon Rapalog induced microtubule anchoring in U2OS cells. (Bottom) Microtubule movement is reduced after Rapalog induced anchoring to the plasma membrane. This video complements Fig. S3.

Supplementary Movie 9. Global microtubule anchoring stops TRIM46 fluctuations. This video complements Fig S3. Scale bar 10 µm.

Supplementary Movie 10. Global microtubule anchoring stops Kinesin-1 fluctuations. This video complements Fig. 2.

Supplementary Movie 11. Light induced local microtubule anchoring directs the selective transport of Kinesin-1 in stage 2 neuron. This video complements Fig. 2.

Supplementary Movie 12. Local Rac1 activation induces anterograde movement of microtubule network in stage 2 neuron. This video complements Fig. 3.
